# Fabrication of Poly Dopamine@poly (Lactic Acid-Co-Glycolic Acid) Nanohybrids for Cancer Therapy via a Triple Collaboration Strategy

**DOI:** 10.3390/nano13091447

**Published:** 2023-04-24

**Authors:** Yunhao Li, Yujuan Gao, Zian Pan, Fan Jia, Chenlu Xu, Xinyue Cui, Xuan Wang, Yan Wu

**Affiliations:** 1 Department of Medicine, Li Ka Shing Faculty of Medicine, University of Hong Kong, Hong Kong, China; yunhaoli@connect.hku.hk; 2 Department of General Surgery, Peking Union Medical College Hospital, Peking Union Medical College, Chinese Academy of Medical Sciences, Beijing 100730, China; 3 CAS Key Laboratory for Biomedical Effects of Nanomaterials and Nanosafety, National Center for Nanoscience and Technology, No. 11 First North Road, Zhongguancun, Beijing 100190, China; gaoyj20211@nanoctr.cn (Y.G.); panza2019@nanoctr.cn (Z.P.); jiaf2018@nanoctr.cn (F.J.); xucl2022@nanoctr.cn (C.X.); cuixy@nanoctr.cn (X.C.); 4 University of Chinese Academy of Sciences, Beijing 100049, China

**Keywords:** nanohybrids, poly dopamine, antiangiogenesis, photothermal therapy, breast cancer

## Abstract

Breast cancer is a common malignant tumor among women and has a higher risk of early recurrence, distant metastasis, and poor prognosis. Systemic chemotherapy is still the most widely used treatment for patients with breast cancer. However, unavoidable side effects and acquired resistance severely limit the efficacy of treatment. The multi-drug combination strategy has been identified as an effective tumor therapy pattern. In this investigation, we demonstrated a triple collaboration strategy of incorporating the chemotherapeutic drug doxorubicin (DOX) and anti-angiogenesis agent combretastatin A4 (CA4) into poly(lactic-co-glycolic acid) (PLGA)-based co-delivery nanohybrids (PLGA/DC NPs) via an improved double emulsion technology, and then a polydopamine (PDA) was modified on the PLGA/DC NPs’ surface through the self-assembly method for photothermal therapy. In the drug-loaded PDA co-delivery nanohybrids (PDA@PLGA/DC NPs), DOX and CA4 synergistically induced tumor cell apoptosis by interfering with DNA replication and inhibiting tumor angiogenesis, respectively. The controlled release of DOX and CA4-loaded PDA@PLGA NPs in the tumor region was pH dependent and triggered by the hyperthermia generated via laser irradiation. Both in vitro and in vivo studies demonstrated that PDA@PLGA/DC NPs enhanced cytotoxicity under laser irradiation, and combined therapeutic effects were obtained when DOX, CA4, and PDA were integrated into a single nanoplatform. Taken together, the present study demonstrates a nanoplatform for combined DOX, CA4, and photothermal therapy, providing a potentially promising strategy for the synergistic treatment of breast cancer.

## 1. Introduction

Breast cancer is one of the most common and invasive malignancies in women [1]. While significant progress has been made in the early diagnosis of breast cancer in recent years, there are still unovercome limitations in current breast cancer therapy modalities, such as multi-drug resistance and dose-limiting side effects [2]. Many clinical cases usually show poor outcomes and efficacy to chemotherapy due to rapidly acquired resistance and distant metastases [3,4,5]. Therefore, new therapeutic strategies, such as combination therapy, which can sensitize tumor cells to chemotherapy, hold great promise for overcoming the current challenges in breast cancer treatment [6,7,8,9].

The combination of two or more therapeutic and diagnostic drugs to form a drug delivery strategy for a nanocarrier platform has great potential and will revolutionize future cancer treatment [10,11]. The strategy of the combination therapy with two or more drugs is a feasible method to overcome the shortcomings of single-drug resistance, low efficacy, high toxicity, and limited clinical application [12,13]. Combination therapy regimens, especially nano-photothermal chemotherapy, have been extensively studied to obtain their synergistic therapeutic effects [14,15,16,17]. Photothermal therapy (PTT) is an effective non-invasive treatment strategy for breast cancer that uses light-absorbing materials to convert light energy into local thermal therapy to ablate tumor tissues and has been widely studied [18].

Up to now, various photothermal-responsive nanoparticles have been shown to be useful for tumor therapy [19,20,21]. Among them, poly(dopamine) (PDA) nanoparticles have shown great advantages in tumor therapy [21]. Dopamine (DA) plays the role of an information transmitter in the brain, which is widely distributed in the human body. DA can self-polymerize and form surface-attached PDA (Figure S1A) films or nanoparticles on a variety of materials [22,23,24,25,26]. PDA nanoparticles have strong near-infrared (NIR) absorption and high photothermal conversion efficiency (40%) and have recently emerged as potential photothermal therapeutic agents for cancer therapy in vivo. Unlike widely used metal- and carbon-based photothermal materials, PDA nanoparticles have excellent biocompatibility, good photothermal stability, negligible cytotoxicity, and can greatly reduce inflammatory reactions [27,28,29]. In addition, PDA nanoparticles have rich aromatic rings on their surface, which can load chemical drugs on their surface through π–π stacking and/or hydrogen bonding [30,31]. They also have chemically active catechol and quinone groups on their surfaces to facilitate further modifications for other functions, such as enhanced blood circulation or cell targeting [32].

As a drug delivery system, polymer nanoparticles have the great advantage of delivering higher concentrations of chemotherapy drugs to the tumor site, thereby reducing systemic toxic effects [33,34]. Poly(lactic-co-glycolic acid) (PLGA) is one of the most successfully applied biodegradable polymers because its hydrolysis can yield metabolic monomers, lactic acid, and glycolic acid (Figure S1B). Because both monomers are endogenous and easily metabolized by the body through the tricarboxylic acid cycle, the systemic toxicity of PLGA for drug delivery or biomaterial applications is minimal [35]. PLGA has received extensive attention due to its good biocompatibility and biodegradability [36].

Combretastatin A4 (CA4) is a natural cis-stilbene derivative that has been used as a tumor vascular targeting agent in cancer therapy [37]. It has been demonstrated that CA4 exhibits anticancer effects through multiple pathways. As a tumor vascular targeting agent, CA4 can inhibit the expression of vascular endothelial growth factor [38]. In addition, CA4 can strongly inhibit tubulin polymerization by binding to colchicine binding sites, reduce mitochondrial oxygen consumption, and induce the apoptosis of cancer cells through cleavage and the activation of caspase 3 and 9 proteins [39,40,41]. However, the hydrophobic nature of CA4 limits its clinical application, and the single-agent treatment mode has significant limitations. Therefore, the encapsulation of CA4 into nano-drug delivery systems for synergistic anti-tumor therapy has attracted much attention in recent years [42,43,44].

Doxorubicin (DOX) is a traditional primary chemotherapy drug that destroys DNA by embedding anthracyclines’ moiety and inhibiting DNA topoisomerase II [45]. DOX has been widely used as an anti-tumor drug in clinical practice [46]. However, its application, such as in limited dosing regimens and narrow therapeutic windows, is severely hampered by its severe side effects on normal tissues. Therefore, the controlled release of DOX to targeted tumor tissues is an urgent clinical challenge that needs to be addressed.

**Scheme 1:**
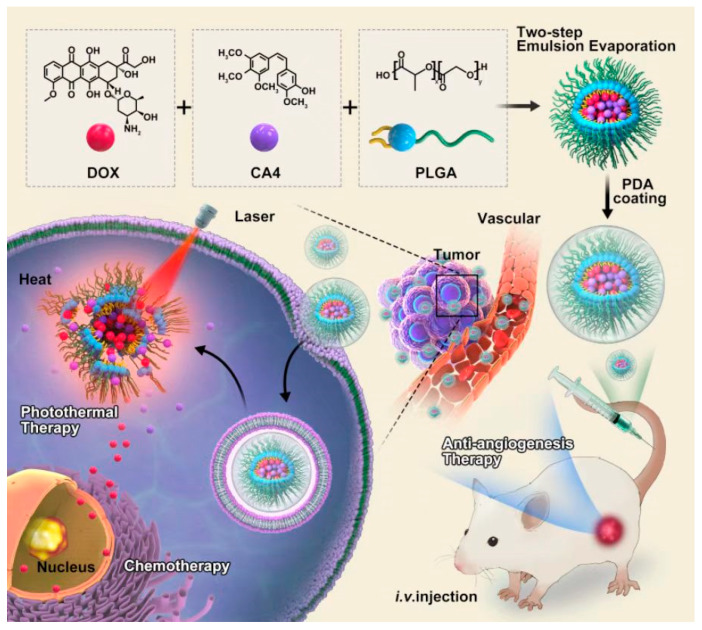
Schematic illustration of the assembly process and the antitumor mechanisms for chemotherapy, antiangiogenesis, and photothermal therapy in breast cancer of PDA@PLGA/DC nanohybrids.

Herein, we developed a unique PDA@PLGA co-delivery nanohybrid system for highly effective combination therapy. The multi-functional nanohybrids (NPs) were simultaneously loaded into PLGA NPs with the hydrophilic chemotherapeutic drug DOX and hydrophobic antiangiogenic agent CA4 through an improved double emulsion method, and then a photothermal treatment was carried out through a self-assembly modified PDA shell. DOX and CA4-dual-loaded PDA@PLGA-co-delivered NPs (PDA@PLGA/DC NPs) showed a tendency to accumulate within tumors and could be internalized by tumor cells to release drugs in acidic organelles. DOX and CA4 synergistically induce the apoptosis of tumor cells by disrupting DNA replication with DOX and inhibiting tumor vasculature with the antiangiogenic effect of CA4. Near-infrared (NIR) laser irradiation can trigger the release of DOX and CA4 from PDA@PLGA/DC NPs and elevate the temperature of the PDA@PLGA/DC NPs’ aqueous solution. The results of in vitro experiments demonstrated that PDA@PLGA/DC NPs combined with laser irradiation could significantly improve the killing efficiency of 4T1 cells and enhance the photocytotoxicity. In addition, in vivo experiments revealed that PDA@PLGA/DC NPs with NIR laser irradiation had highly synergistic anticancer effects on 4T1 tumor-bearing mice. Combining photothermal therapy with DOX and CA4 in a biocompatible nanoplatform ultimately achieved tumor ablation with minimal side effects and will be beneficial for improving the effectiveness of breast cancer therapy. A detailed illustration of the combined PDA@PLGA/DC nanohybrids co-delivery system with NIR laser irradiation is exhibited in Scheme 1.

## 2. Experimental Section

### 2.1. Materials

PLGA (poly-lactide/glycolide = 75:25, MW = 20,000 Da) was obtained from Daigang BIO Engineer Limited Co. (Jinan, China). Dopamine hydrochloride was acquired from J&K Chemical Ltd. (Beijing, China). Doxorubicin hydrochloride salt (DOX) was purchased from Beijing HuaFeng Co., Ltd. (Beijing, China). Combretastatin A4 (CA4) was produced from Hangzhou Great Forest Biomedical Co., Ltd. (Hangzhou, China). Sodium cholate was from Sigma-Aldrich (St. Louis, MO, USA). Peniciline-streptomycin, Dulbecco’s modified Eagle’s medium (DMEM), and trypsin were obtained from Gibco BRL (Grand Island, NY, USA). Fetal bovine serum (FBS) was purchased from Nanjing Wisdom Biotechnology Co., Ltd. (Nanjing, China). Hoechst33342 and LysoTracker Red DND-99 were obtained from Thermo Fisher Scientific (Waltham, MA, USA). Cell Counting Kit-8 (CCK-8) was purchased from Dojindo Molecular Technologies (Tokyo, Japan). Unless otherwise stated, all other chemicals and solvents were of analytical grade and used after the removal of water. Ultra-pure water (deionized water) was from a Milli-Q water system (Millipore, Bedford, MA, USA).

### 2.2. Preparation of PDA@PLGA/DC NPs

Blank PLGA nanoparticles (PLGA NPs) were prepared by self-assembly method based on nano-precipitation. Briefly, 10 mg of PLGA was dissolved in 1 mL of dimethyl sulfoxide (DMSO) to form 10 mg/mL PLGA polymer solution. Then, 1 mL of the above PLGA solution was added to 5 mL of aqueous solution under magnetic stirring and stirred for another 30 min at room temperature.

PLGA NPs were obtained by centrifugation for 20 min at 10,000 rpm and washed three times with deionized water.

DOX-loaded PLGA nanoparticles (PLGA/D NPs) were fabricated by double emulsion method. Briefly, PLGA was dissolved in dichloromethane to form 10 mg/mL polymer solution. DOX aqueous solution (2.0 mg/mL) with a volume of 50–250 μL was added to the 1 mL polymer solution. Primary water-in-oil (W_1_/O) emulsion was obtained by ultrasonic treatment at 30 W for 5 min. The resulting emulsion was slowly added to 4 mL of 1% (*w*/*v*) sodium cholate solution and sonicated for 5 min (30 W) to make a water-in-oil (W_1_/O/W_2_) double emulsion.

After evaporation of the solvent in vacuum, PLGA/D NPs were obtained via centrifugation at 12,000 rpm for 10 min and washed three times using deionized water.

PLGA nanoparticles containing CA4 (PLGA/C NPs) were prepared by emulsion/solvent evaporation technique. In brief, 10 mg of PLGA polymer was dissolved in 1 mL of dichloromethane, and CA4 (dissolved in DMSO) was added at mass ratios from 20/1 to 100/1 (PLGA/CA4). The mixture was emulsified for 10 min at room temperature by sonication (10 W) in 10 mL 1% (*w*/*v*) sodium cholate solution in an external ice bath. The solvent was evaporated and eliminated under vacuum. Finally, PLGA/C NPs were obtained via centrifugation at 12,000 rpm for 10 min at room temperature and washed three times with deionized water.

PLGA nanoparticles loaded with DOX/CA4 (PLGA/DC NPs) were constructed by an improved double-emulsion technique. In brief, 10 mg of PLGA was dissolved in a mixture of dichloromethane (1 mL) and different volumes of DOX (2 mg/mL) aqueous solution. Then it was emulsified via sonication in an external ice bath for 5 min (10 W). Then 4 mL of 1% (*w*/*v*) sodium cholate was added and stirred at room temperature for 10 min. At the same time, different volumes of 10 mg/mL CA4 (dissolved in DMSO) dispersed in dichloromethane were slowly added and then emulsified through sonication in an external ice bath for 5 min (30 W). Then we slowly added 10 mL of 1% (*w*/*v*) sodium cholate into the mixture and stirred for 10 min. The solvent was removed under reduced pressure using a rotary evaporator and the PLGA/DC NPs were recovered through centrifugation for 10 min at 12,000 rpm at room temperature and washed three times with deionized water.

Polydopamine (PDA) coating was formed on the surface of PLGA/DC NPs by adding 2 mg of PLGA/DC NPs to 1 mL of dopamine hydrochloride solution in tris buffer (10 mM, pH 8.5) and rotating for 24 h at room temperature. PDA@PLGA/DC NPs were obtained by centrifugation for 15 min at 15,000 rpm and purified by repeated washing with deionized water. The dopamine concentration was fixed at 1 mg/mL and left unchanged unless otherwise stated. Other PDA@PLGA NPs, PDA@PLGA/D NPs, and PDA@PLGA/C NPs were obtained by the same procedure.

### 2.3. Physicochemical Characterizations of PDA@PLGA/DC NPs

The mean particle size, polydispersity index (PDI), zeta potential, and stability of NPs were measured via dynamic light scattering (DLS) using a Zetasizer Nano-series Nano ZS (Malvern Instruments Ltd., Malvern, UK). Detections were performed at 25 °C with a wavelength of 633 nm and constant scattering angle of 90°, and samples were appropriately diluted with deionized water before testing. The morphology of NPs was observed by transmission electron microscopy (TEM) (Hitachi Corporation, Tokyo, Japan). Uv–vis adsorption was determined with a LAMBDA 650 (PerkinElmer, Fremont, CA, USA). The amount of DOX and CA4 encapsulated in NPs was determined by UV-vis spectrophotometer at 480 nm and 294 nm, respectively, and calculated by standard curves.

Encapsulation efficiency (EE) and loading capacity (LC) were defined as follows:

EE = (drug loading weight)/(weight of the drug initially added) × 100%

LC = (drug loading weight)/(total weight of nanohybrids and drugs) × 100%

### 2.4. Photothermal Effects of PDA@PLGA/DC NPs

PDA can absorb NIR light and convert it into heat, which can be used to kill tumor cells and accelerate drug release. To evaluate the effect of NP concentration on the photothermal efficiency, PDA@PLGA/DC NPs with different concentrations (0.25 to 150 μg/mL) of phosphate buffer saline (PBS) (pH = 7.4) solution were added to the centrifuge tubes. The centrifuge tubes were irradiated with an 808 nm laser (Beijing Laserwave Optoelectronics Technology Co., Ltd., Beijing, China) for 5 min at a power density of 1 W/cm^2^, and the temperature changes was measured. PBS solution was used as the control group. Maximum regional temperature and infrared thermogram were obtained by an infrared thermal imaging camera (Ti27, Fluke, Everett, WA, USA).

### 2.5. In Vitro Release Behavior

The in vitro release of DOX and CA4 from PDA@PLGA/DC NPs was determined through dialysis method. Lyophilized PDA@PLGA/DC NPs were dispersed in 3 mL PBS and transferred to dialysis bags (the molecular weight cutoff was 3500 Da). The compact dialysis bags were incubated in 30 mL PBS solution (pH 5.2, 7.4) at 37 °C, with or without 808 nm continuous-wave diode laser (Daheng Science & Technology, Beijing, China) irradiation for 5 min at 1 W/cm^2^ (with a laser spot diameter of 4 mm). Agitation was carried out at 100 rpm/min through a water bath thermostatic oscillator. At various time intervals, 1 mL of the supernatant was removed and replaced with 1 mL of fresh buffer. The amount of DOX and CA4 released from the media was measured by UV-vis spectrophotometry as mentioned above. The cumulative amount of drug released was calculated, and the percentage of drug released by NPs was plotted against time.

### 2.6. Cellular Uptake In Vitro

The 4T1 cells were seeded on borosilicate chambered coverglass at a density of 2 × 10^5^ cells per well, cultured in DMEM medium containing 10% FBS, and incubated for 24 h at 37 °C in a 5% CO_2_ incubator. The original medium was then replaced with 200 μL of complete medium containing PDA@PLGA/DC NPs. After incubation for 0.5 h, 6 h, and 6 h+NIR, respectively, cells were washed three times with PBS and LysoTracker Red DND-99 was used to label lysosomes according to the manufacturer’s instructions. The nucleus was stained via Hoechst 33342. Cell images were taken using a laser scanning confocal microscope (CLSM, Carl Zeiss, Boston, MA, USA). After incubation of PDA@PLGA/DC NPs with 4T1 cells for 2 h, the cells were irradiated with 808 nm laser at 1 W/cm^2^ for 5 min and incubated for another 2 h to further investigate the photo-thermal effects on cell uptake.

### 2.7. Cytotoxicity Assay

The 4T1 cells were cultured in DMEM medium supplemented with 10% FBS, 1% penicillin, and 1% streptomycin and incubated at 37 °C with 5% CO_2_. To evaluate the cytotoxicity of the NPs, 4T1 cells were seeded in 96-well plates at a density of 2 × 10^4^ cells per well and incubated for 12 h at 37 °C in 5% CO_2_. Then, the original medium was replaced with 200 μL of various concentrations of PDA@PLGA NPs, PDA@PLGA NPs+NIR, free DOX, PDA@PLGA/D NPs+NIR, free CA4, PDA@PLGA/C NPs+NIR, and PDA@PLGA/DC NPs and PDA@PLGA/DC NPs+NIR (DOX:CA4, 4:1, mass ratio) in fresh medium for 24 h. Cell viability was assessed by CCK-8 assay according to the manufacturer’s instructions (Dojindo, Kumamoto, Japan). Subsequently, we evaluated the PTT effect of different formulations of NPs. The 4T1 cells were seeded in 96-well plates at a density of 5 × 10^3^ cells per well and cultured for 12 h. The original culture medium was replaced with 200 μL of fresh culture medium containing equivalent concentrations of PDA@PLGA NPs, PDA@PLGA/D NPs, PDA@PLGA/C NPs, and PDA@PLGA/DC NPs. After 12 h of culture, the cells were treated with 808 nm laser irradiation at a power density of 1 W/cm^2^ for 5 min. The treated cells were cultured for another 24 h and the cell viability of each group was detected using CCK-8 method.

### 2.8. Experimental Animals

Healthy female BALB/c mice (6 to 7 weeks old) were purchased from Beijing Vital River Laboratory Animal Technology Co., Ltd. (Beijing, China). All experimental animals were operated on in accordance with the Guide for the Care and Use of Laboratory Animals of the National Center for NanoScience and Technology, approved by the Institutional Animal Care and Use Committee (IACUC) (ethic approval number: NCNST-220902), and in accordance with the relevant laws for laboratory animals in China.

### 2.9. In Vivo Imaging

For in vivo imaging, 100 μL IR780-loaded PDA@PLGA/DC NPs (0.7 mg/kg, IR780) were injected intravenously into 4T1 tumor-bearing mice. Fluorescence imaging was performed using CRI Maestro in vivo optical imaging system at 0, 2, 4, 8, 12, and 24 h after injection.

### 2.10. In Vivo Antitumor Treatments

When the tumor volume reached about 100 mm^3^, 4T1 tumor-bearing mice were randomly divided into five treatment groups with 5 mice in each group. Saline, PDA@PLGA NPs+NIR, PDA@PLGA/C NPs, PDA@PLGA/D NPs, and PDA@PLGA/DC NPs+NIR were injected through the tail vein. Tumor volume and body weight were recorded every other day. For the laser irradiation group, the tumor of mice was irradiated with 808 nm laser at 1 W/cm^2^ for 10 min at 24 h after injection. Tumor size was measured with a vernier caliper and tumor volume was calculated (tumor volume = tumor length × tumor width^2^/2). After 15 days, the mice were sacrificed, and the tumors were removed and weighed.

### 2.11. Biochemical Analysis and Histological

The major organs (heart, lung, liver, spleen, and kidney) of mice treated with different formulations were collected, fixed with 4% (*w*/*v*) paraformaldehyde, stained with hematoxylin and eosin (H&E), and visualized by light microscopy. After treatment with various drug formulations, serum from slaughtered animals was harvested for blood biochemical analysis, and data were obtained from Charles River Laboratories (Beijing, China).

### 2.12. Statistic Analysis

Data are presented as mean ± standard deviation (SD) of at least three independent experiments. Statistical differences were analyzed using the Student’s *t*-test and are presented as error bars. 

## 3. Results and Discussion

### 3.1. Construction and Characterization of PDA@PLGA/DC NPs

Synergistic drug combinations are commonly used to improve efficacy and reduce side effects. Previous work has shown that two chemotherapeutic agents prepared in a nanoplatform show synergistic inhibitory effects against multiple types of cancer [9]. The double emulsion method is the most reliable method to prepare multiple lotions, especially the three-component system (W/O/W). The first step is to prepare a W/O lotion with a lipophilic emulsifier and then add the lotion to the water phase containing a hydrophilic emulsifier to prepare a W/O/W lotion. Therefore, we incorporated the chemotherapeutic drug DOX and the antiangiogenic agent CA4 into a PDA@PLGA-based nanoplatform for combined chemotherapy and photothermal therapy. The DOX- and CA4-loaded PDA@PLGA nanohybrids (PDA@PLGA/DC NPs) were constructed using a modified double-emulsification technology, in which the PDA@PLGA-prepared nanoparticles were simultaneously encapsulated with DOX and CA4 (Scheme 1).

**Figure 1:**
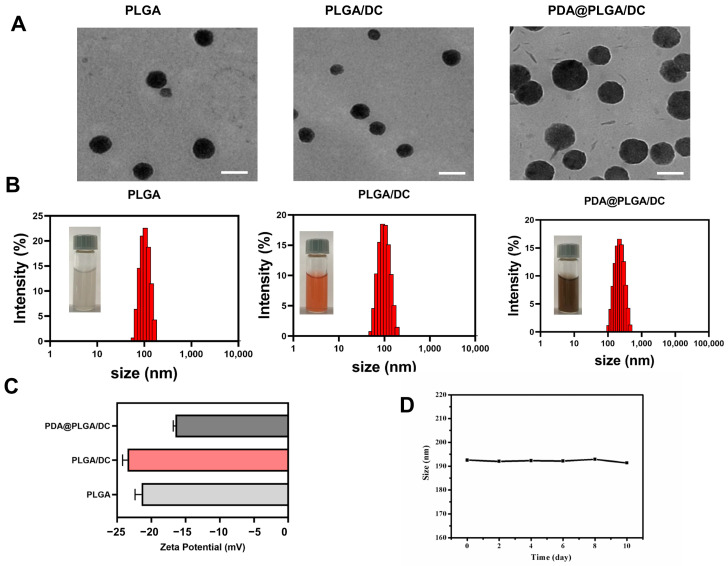
Characterizations of PDA@PLGA/DC NPs. (**A**) TEM. The scale bar is 200 nm. (**B**) Particle size distribution of PLGA NPs, PLGA/DC NPs, and PDA@PLGA/DC NPs. (**C**) Zeta potentials of PLGA NPs, PLGA/DC NPs, and PDA@PLGA/DC NPs. (**D**) The average size of PDA@PLGA/DC NPs after being maintained in PBS for different time intervals. Abbreviations: PLGA, PLGA NPs; PLGA/DC, PLGA/DC NPs; PDA@PLGA/DC, PDA@PLGA/DC NPs.

The morphology of the PLGA NPs, PLGA/DC NPs, and PDA@PLGA/DC NPs were characterized by TEM. The PDA@PLGA/DC NPs were dispersed as individual particles with a typical spherical shape and good dispersion (Figure 1A). The mean hydrodynamic diameter and surface charge of the NPs obtained in PBS were determined by DLS (Figure 1B,C). The average particle size of the PLGA NPs was 100.4 nm (PDI: 0.044), while the average particle size of the PLGA/DC NPs was slightly reduced by 94.7 nm (PDI: 0.08), which can be attributed to the interaction of the PLGA and drugs. When the surface was coated with PDA, the PDA@PLGA/DC NP solution could be seen to be a transparent black-brown color, and the average particle size of the PDA@PLGA/DC NPs was 190.4 nm (PDI, 0.108). TEM also revealed that the particle size increased significantly after PDA coating, and the spherical particles were more regular. The NPs’ size distribution was narrow (PDI < 0.2). The zeta potentials of the PLGA NPs, PLGA/DC NPs, and PDA@PLGA/DC NPs were −21.4, −23.5, and −16.5 mV, respectively. Compared with the PLGA/DC NPs, the zeta potential of the PDA@PLGA/DC NPs changed from −23.5 mV to −16.5 mV, and the changes in the zeta potential further indicated the successful encapsulation of PDA (Figure 1C). The characteristic absorption peaks of the -NH_2_ functional groups were observed at 3355 cm^−1^ and 3325 cm^−1^ PDA@PLGA NPs, which also supported the successful coating of the PDA (Figure S2). In addition, we evaluated the stability of the PDA@PLGA/DC NPs. As shown in Figure 1D, no significant changes in size were observed after 10 days of storage at 37 °C.

The encapsulation efficiency and drug loading of NPs are very important for in vitro and in vivo studies. Therefore, this study investigated the effect of different dosage forms on the drug encapsulation efficiency and drug loading. Previous studies have shown that the polymer-to-drug ratio is critical for evaluating the drug encapsulation efficiency and drug loading [44]. Therefore, the effect of the polymer/drug mass ratio on the drug entrapment efficiency and drug loading was evaluated. As shown in Tables S1 and S2, when the mass of PLGA was 10 mg and the dosages of DOX and CA4 were different masses (100 μg~500 μg), with the increase in DOX dosage, the drug encapsulation and loading of DOX first increased and then decreased. The drug encapsulation efficiency of CA4 gradually decreased. According to the different encapsulation effects of PLGA on hydrophobic and hydrophilic drugs, a mass ratio of PLGA, DOX, and CA of 100:4:1 was selected as the optimal ratio for subsequent studies, which had a high encapsulation efficiency and drug loading. These findings suggested that PLGA-based drug-loaded nanoparticles could form homogeneous and stable nanostructures with appropriate sizes and negative surface charges. The nanoparticles showed great advantages in terms of stability, drug-loading efficiency, and hydrophilic and hydrophobic drug co-delivery.

### 3.2. Photothermal Heating Effect

**Figure 2:**
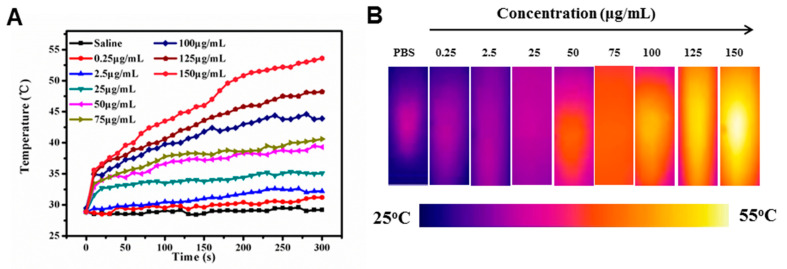
(**A**) Temperature curves of saline and PDA@PLGA/DC NPs with different concentrations exposed to the 808 nm laser at a power density of 1 W/cm^2^ for 5 min. (**B**) The infrared thermal image of PBS and PDA@PLGA/DC NPs with different concentrations exposed to the 808 nm laser at a power density of 1 W/cm^2^ for 5 min.

The photothermal effect of the PDA@PLGA/DC NPs was evaluated by detecting the temperature change by in vitro irradiation with an 808 nm near-infrared laser. As shown in Figure 2A,B, the solution temperature of the PDA@PLGA/DC NPs increased rapidly under 808 nm laser irradiation (1.0 W/cm^2^), and the solution temperature exceeded 54 °C within 5 min when the concentration of the PDA@PLGA/DC NPs was 150 µg/mL. In contrast, the PBS solution was kept below 37 °C and only increased to 29 °C under the same laser irradiation (Figure 2A,B). Such an excellent photothermal conversion performance of PDA@PLGA/DC NPs has been shown in many previous studies. PDA can also act as a photothermal agent, and PDA@PLGA/DC NPs have significant near-infrared absorption and an excellent photothermal conversion efficiency [47,48]. The effects of the PDA@PLGA/DC NPs with concentrations ranging from 0.25 to 150 μg/mL on the photothermal efficiency were detected by determining the temperature changes via 808 nm laser irradiation. The photothermal efficiency of the PDA@PLGA/DC NPs was concentration dependent, and the temperature of the PDA@PLGA/DC NPs increased with increasing concentrations (Figure 2A,B). Thus, the excellent photothermal efficiency of the PDA@PLGA/DC NPs revealed above shows the great potential of using PDA@PLGA/DC NPs as a thermal therapy agent.

### 3.3. In Vitro Drug Release from PDA@PLGA/DC NPs

**Figure 3:**
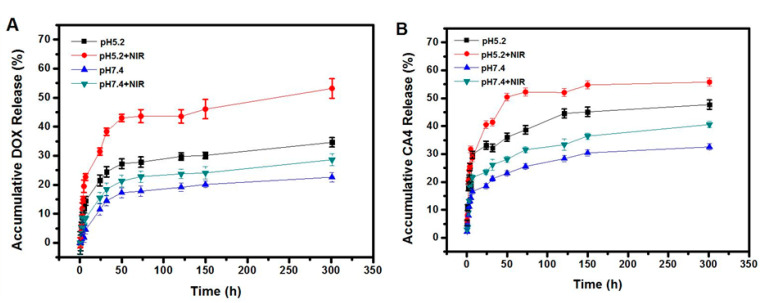
(**A**) DOX and (**B**) CA4 release profiles from PDA@PLGA/DC NPs at different pHs with or without NIR laser irradiation (808 nm, 1 W/cm^2^).

The manner of DOX and CA4’s release from the PDA@PLGA/DC NPs was explored in vitro at pHs of 7.4 and 5.2, 37 °C, with or without near-infrared laser irradiation. Figure 3A,B revealed that the amounts of DOX and CA4 released from the PDA@PLGA/DC NPs demonstrated a pH and NIR laser-irradiation-dependent release pattern. Previous studies have shown that more than 30% of the drug is released from nanoparticles within 8 h [23]. It is interesting to note that the modification of the PDA on the NPs’ surface markedly inhibited the initially uncontrollable release of the drug. Interestingly, the cumulative release of DOX and CA4 was significantly promoted by 808 nm NIR irradiation at different time points and pH values. The reason for the enhanced DOX and CA4 release under NIR laser irradiation might be that the increase in temperature weakened the interaction between the DOX, CA4, and NPs. The results indicated that the combined photothermal therapy significantly increased the sensitivity of DOX and CA4. In addition, the release effects of DOX and CA4 also demonstrated pH responsiveness. The pH-sensitive DOX and CA4 release of PDA@PLGA/DC NPs was mainly attributed to the PDA polymer coating being partially stripped from the surface of PLGA NPs in acidic media, thus causing apparent drug release [49]. Under the treatment of PBS at pH 5.2 and laser irradiation, the cumulative drug release percentage of DOX and CA4 could reach about 53% and 55%, respectively. DOX and CA4 release triggered by pH-dependent and near-infrared laser irradiation could effectively enhance the synergistic chemotherapy, anti-tumor angiogenesis, and photothermal therapy effects.

### 3.4. In Vitro Synergy Therapy

**Figure 4:**
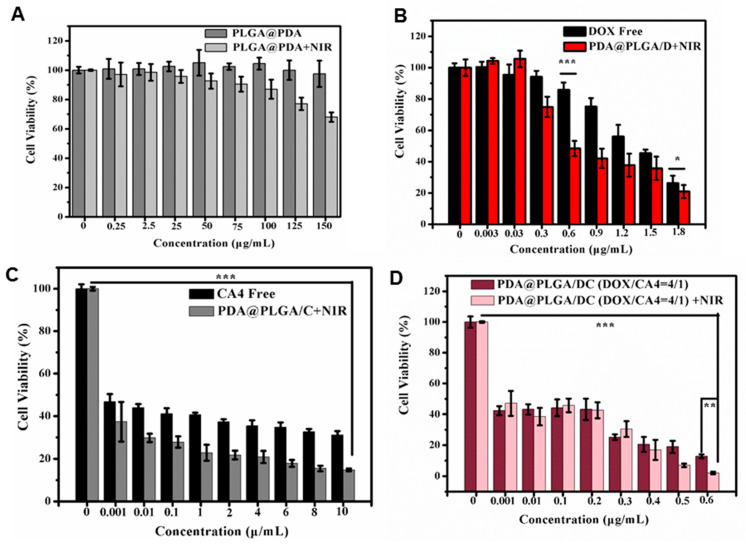
Cell viability of 4T1 cells treated with different formulations. (**A**) Cell viability of 4T1 incubated with different concentrations of PDA@PLGA NPs and PDA@PLGA NPs+NIR for 24 h. (**B**) Cell viability of 4T1 incubated with different concentrations of free DOX and PDA@PLGA/D NPs+NIR for 24 h. (**C**) Cell viability of 4T1 incubated with different concentrations of free CA4 and PDA@PLGA/C NPs+NIR for 24 h. (**D**) Cell viability of 4T1 incubated with different concentrations of PDA@PLGA/DC NPs and PDA@PLGA/DC NPs+NIR for 24 h. Abbreviations: PLGA@PDA, PLGA@PDA NPs; PLGA@PDA+NIR, PLGA@PDA NPs+NIR; PDA@PLGA/D+NIR, PDA@PLGA/D NPs+NIR; PDA@PLGA/C+NIR, PDA@PLGA/C NPs+NIR; PDA@PLGA/DC, PDA@PLGA/DC NPs; PDA@PLGA/DC+NIR, PDA@PLGA/DC NPs+NIR. (* *p* < 0.05, ** *p* < 0.01 and *** *p* < 0.001).

At present, although the thermal ablation of tumor cells in PTT is considered to be a promising method for the treatment of localized tumors, it is difficult to treat metastatic tumors well. In addition, tumor heterogeneity and complexity also require combined strategies to surmount these limitations. Therefore, the combination of PTT with chemotherapy, photodynamic therapy, gene therapy, and other treatment methods has shown to have better therapeutic effects [50,51,52]. Therefore, to assess the combined effect of PTT of our prepared PDA@PLGA/DC NPs with DOX and CA4, 4T1 cells were treated by different formulations for 24 h and the cell viability was determined by CCK-8 (Figure 4).

To explore the toxicity of blank PDA@PLGA NPs, the potential cytotoxicity of concentrations ranging from 0.25 to 150 μg/mL in 4T1 cells was assessed. As displayed in Figure 4A, when the blank PDA@PLGA NPs’ concentration reached 150 μg/mL, the cell viability remained above 90%, indicating that the blank PDA@PLGA NPs had low cytotoxicity and good compatibility. The blank PDA@PLGA NPs in the 4T1 cells were subjected to NIR laser irradiation at 1 W/cm^2^ for 5 min. As exhibited in Figure 4A, the effect of the photothermal therapy on the tumor cells was evident in a concentration-dependent cytotoxic manner. The cell viability was 70% at a concentration of 150 μg/mL, and 100 μg/mL (90% cell viability) was used for the subsequent laser-irradiated cell experiments. The results for the cell survival under the conditions of free DOX, PDA@PLGA/D NPs+NIR, free CA4, and PDA@PLGA/C NPs+NIR at different drug concentrations are shown in Figure 4B,C. It can be observed that with the increase in drug concentration, the cell survival rate in the single-drug DOX group gradually decreased. However, the single CA4 group achieved a 50% cytotoxic effect at a very low drug dose (0.001 μg/mL). When combined with the photothermal action, that is, PDA@PLGA/D NPs+NIR and PDA@PLGA/C NPs+NIR, the groups had an obvious synergistic effect. When the DOX concentration was 0.6 μg/mL, the synergistic effect was very good. As can be seen in the dual-drug combination-PDA@PLGA/DC NPs’ group, even without the photothermal effect, the PDA@PLGA/DC NPs’ group still had a significant killing effect on tumor cells. With the increase in DOX concentration, the synergistic effect of the dual-drug combination was more evident. In the multifunctional nanohybrids’ group (PDA@PLGA/DC NPs+NIR) (Figure 4D), the dual-drug combination was used to assist the photothermal therapy, which demonstrated a greater efficacy against tumor cells (the cell survival rate was less than 10%).

### 3.5. Intracellular Uptake Assays of PDA@PLGA/DC NPs

As described above, the PDA@PLGA/DC NPs demonstrated evident cytotoxicity. We then explored the intracellular uptake of PDA@PLGA/DC NPs in 4T1 cells. The uptake and intracellular localization of the PDA@PLGA/DC NPs in the 4T1 cells were observed by confocal microscopy via the fluorescence property of DOX. Red fluorescence indicated PDA@PLGA/DC NPs, and green fluorescence indicated lysosomes. The PDA@PLGA/DC NPs were co-incubated with the 4T1 cells for 0.5 h, 6 h, and 6 h+NIR with NIR laser irradiation to detect the intrinsic fluorescence signal of the PDA@PLGA/DC NPs.

**Figure 5:**
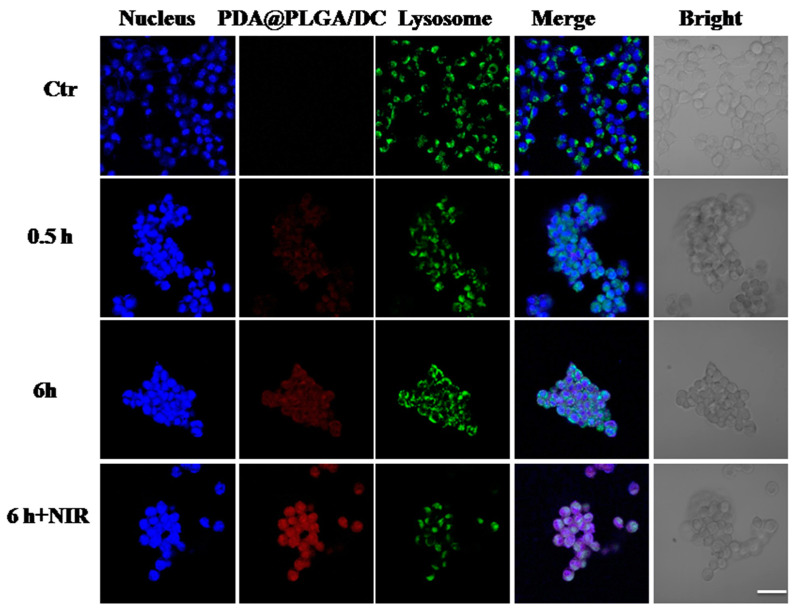
Intracellular localization of PDA@PLGA/DC NPs in 4T1 cells. Cells were incubated with PDA@PLGA/DC NPs for 0.5 h, 6 h, and 6 h+NIR, respectively, and then observed by confocal laser scanning microscopy. Intracellular localization of PDA@PLGA/DC NPs (red) and lysosomes (green) in 4T1 cells. The nucleus (blue) was stained by Hoechst 33342 (Ho.33342). Abbreviations: PDA@PLGA/DC, PDA@PLGA/DC NPs.

As exhibited in Figure 5, with the increase in incubation time, the red fluorescence of the NPs became more and more intense, and there was an obvious overlap with the blue fluorescence of the nucleus at 6 h, indicating that more and more drugs could enter the cells to exert pharmacological effects, and a large number of drugs entered the nucleus to achieve the effect of killing tumor cells. After 6 h of laser irradiation, the green fluorescence signals of the lysosomes were observed to become weak, demonstrating that the temperature of the laser-irradiated NPs damaged the lysosomes. The red fluorescence and blue nuclei almost completely overlapped, further proving that laser irradiation could facilitate the entry of nanoparticles into the nucleus.

### 3.6. Synergistic Therapeutic Effect of PDA@PLGA/DC NPs In Vivo

**Figure 6:**
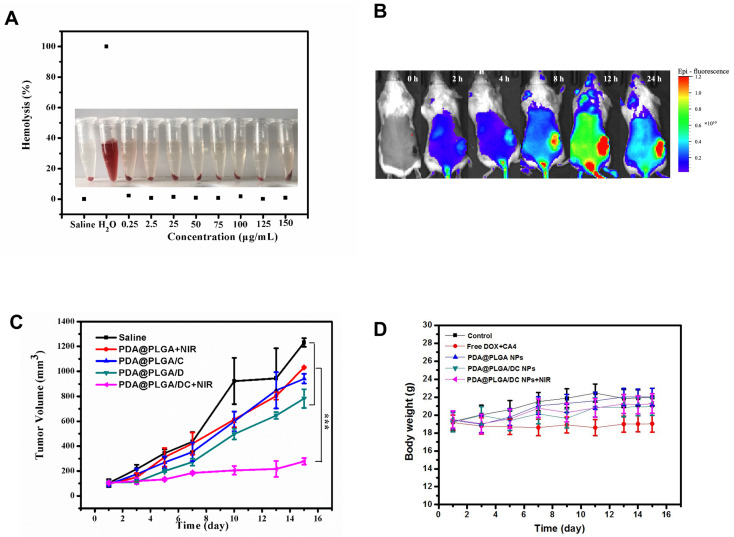
(**A**) Hemolysis assay of with different concentrations of PDA@PLGA/DC NPs using water as a positive control and saline as a negative control. (**B**) In vivo fluorescence images of the PDA@PLGA/DC NPs s in a 4T1 tumor-bearing mice model at different time intervals after intravenous injection. (**C**) Tumor growth curves of mice receiving intravenous injections of different formulations *(n* = 5). (**D**) Body weight changes of 4T1 tumor-bearing mice in the therapeutic period. Abbreviations: PDA@PLGA+NIR, PDA@PLGA NPs+NIR; PDA@PLGA/C, PDA@PLGA/C NPs; PDA@PLGA/D, PDA@PLGA/D NPs; PDA@PLGA/DC+NIR, PDA@PLGA/DC NPs+NIR. ( *** *p* < 0.001).

Prior to the in vivo experiments, we first evaluated the blood compatibility of the PDA@PLGA/DC NPs by hemolysis assays. As expected, the PDA@PLGA/DC NPs failed to drive hemoglobin release from the erythrocytes even at 150 μg/ mL (Figure 6A).

To assess the distribution behavior of PDA@PLGA/DC NPs in vivo, we chose IR780 as a fluorescent marker to package it in PDA@PLGA/DC NPs for whole-body fluorescence imaging in mice, and the mice were intravenously injected with PDA@PLGA/DC/IR780 NPs. The fluorescence signals were detected by an ex/in vivo imaging system (CRi, Woburn, MA, USA) 0, 2, 4, 8, 12, and 24 h after the injection. As illustrated in Figure 6B, the fluorescent signal was clearly distributed in the mice 2 h after the injection. Notably, 8 h after the injection, the fluorescence signals demonstrated an obvious trend at the tumor area. At 12 and 24 h, stronger signals appeared at the tumor site, indicating that the PDA@PLGA/DC NPs were efficiently accumulated at the tumor region.

The anti-tumor activity of the PDA@PLGA/DC NPs+NIR was observed in the 4T1 tumor-bearing mice. The tumor-bearing mice were detected and carried out with saline, PDA@PLGA NPs+NIR, PDA@PLGA/D NPs, PDA@PLGA/C NPs, and PDA@PLGA/DC NPs+NIR. As exhibited in Figure 6C, monotherapy (PDA@PLGA NPs+NIR, PDA@PLGA/D NPs, PDA@PLGA/C NPs) demonstrated very limited antitumor efficacy compared with that of the control group, only achieving 10~30% tumor suppression. However, the dual-drug-loaded NPs combined with the NIR irradiation treatment group (PDA@PLGA/DC NPs+NIR) could induce cell karyolysis and apoptosis and showed a very significant tumor growth inhibition effect. The tumor growth was almost completely inhibited, further indicating that chemical photothermal therapy combined with multimodal anti-tumor therapy could produce effective tumor inhibition.

The body weight of the mice in each treatment group was recorded every 2 days. As shown in Figure 6D, there was no significant weight loss in the different formulation groups, indicating that the multifunctional NPs had good biocompatibility and biosafety for in vivo applications.

### 3.7. Potential Adverse Effects of PDA@PLGA/DC NPs

**Figure 7:**
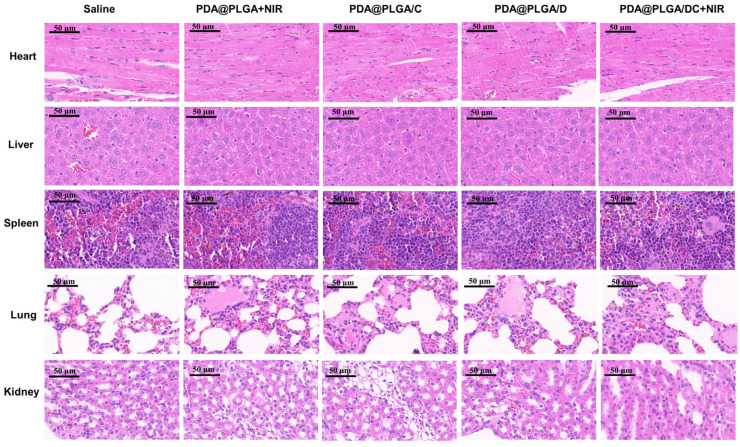
H&E histological analysis of major organs, including heart, liver, spleen, lung, and kidney after treatment with various formulations. Scale bars in all images represent 50 μm. Abbreviations: PDA@PLGA+NIR, PDA@PLGA NPs+NIR; PDA@PLGA/C, PDA@PLGA/C NPs; PDA@PLGA/D, PDA@PLGA/D NPs; PDA@PLGA/DC+NIR, PDA@PLGA/DC NPs+NIR.

The potential in vivo toxicity of NPs in the biomedical field has been the focus of much attention. Thus, we next evaluated the potential side effects of various pharmaceutical preparations on the major organs in mice. H&E staining was used to observe the postmortem histopathology of the main organs (heart, liver, spleen, lung, and kidney) of tumor-bearing mice after treatment with different drug preparations. As shown in Figure 7, there were no significant morphological differences between these treatment groups, which is consistent with previous studies that have shown good tissue and blood compatibility for PDA-modified NPs over a longer period of time [21].

To further verify the efficacy of the PDA@PLGA/DC NPs combined with the laser irradiation group, their serum biochemical indexes were monitored. There were significant differences in some serum biochemical parameters between the tumor-bearing mice and normal mice (control), mainly related to the implantation of tumor xenografts, as shown in Figure S4. After treatment with the PDA@PLGA/DC NPs combined with NIR, the biochemical indicators showed that organ function tended to be normal, consistent with the inhibitory effect on tumor growth. In addition, no additional serum biochemical abnormalities were observed after treatment with PDA@PLGA/DC NPs+NIR, indicating that PDA@PLGA/DC NPs+NIR were safe for tumor therapy. The above results indicate that a nanoplatform integrating chemotherapy, anti-tumor angiogenesis, and photothermal therapy not only has synergistic anti-tumor effects, but also has no significant adverse effects.

## 4. Conclusions

In conclusion, a multifunctional drug-loaded nanoplatform based on PDA@PLGA NPs was successfully designed and constructed for a combination of the chemotherapy drug DOX, antiangiogenesis agent CA4, and photothermal therapy. NPs were fabricated from biocompatible PDA coated with PLGA and loaded with DOX and CA4, using an improved double-emulsification and self-assembly technique. The prepared PDA@PLGA/DC NPs exhibited good dispersion, stability, and remarkable photothermal conversion properties under NIR laser irradiation. In vitro and in vivo experiments showed that the blank PDA@PLGA NPs had minimal cytotoxicity and good biocompatibility. In contrast, PDA@PLGA/DC NPs+NIR significantly improved the therapeutic sensitivity of DOX and CA4 as well as illustrated prominent chemotherapy–hyperthermia synergy under NIR laser irradiation, which might be due to their high drug-loading efficiency, inherent characteristics of photothermal conversion, and the promotion of DOX and CA4 release from PDA@PLGA/DC NPs by NIR laser irradiation. Therefore, a smart nanoplatform based on a PDA@PLGA nanocarrier has brilliant application potential in multimodal combined anti-tumor therapy and is expected to provide a highly transformative approach for breast cancer treatment.

## Data Availability

The data is included in the main text and the supplementary materials.

## Supplementary Materials

The following supporting information can be downloaded at: https://www.mdpi.com/2079-4991/13/9/1447/s1, Figure S1: The structure PDA (A) and PLGA (B); Table S1: Influence of the mass of DOX on encapsulation efficiency (EE) and loading content (LC) of PDA@PLGA/DC NPs; Table S2: Influence of the mass of CA4 on encapsulation efficiency (EE) and loading content (LC) of PDA@PLGA/DC NPs; Figure S2: FT-IR spectra of PDA@PLGA NPs, PLGA NPs and PDA NPs; Figure S3: Temperature variation curves of PDA@PLGA/DC NPs at various power intensities with the same concentration at 150 μg/mL; Figure S4: Serum biochemical examination of drug-loaded nanoparticles treated mice by saline, free DOX+CA4 and PDA@PLGA/DC NPs+NIR.
