# Correction: Li et al. Fabrication of Poly Dopamine@poly (Lactic Acid-Co-Glycolic Acid) Nanohybrids for Cancer Therapy via a Triple Collaboration Strategy. *Nanomaterials* 2023, *13*, 1447

**DOI:** 10.3390/nano14020200

**Published:** 2024-01-16

**Authors:** Yunhao Li, Yujuan Gao, Zian Pan, Fan Jia, Chenlu Xu, Xinyue Cui, Xuan Wang, Yan Wu

**Affiliations:** 1 Department of Medicine, Li Ka Shing Faculty of Medicine, University of Hong Kong, Hong Kong, China; yunhaoli@connect.hku.hk; 2 Department of General Surgery, Peking Union Medical College Hospital, Peking Union Medical College, Chinese Academy of Medical Sciences, Beijing 100730, China; 3 CAS Key Laboratory for Biomedical Effects of Nanomaterials and Nanosafety, National Center for Nanoscience and Technology, No. 11 First North Road, Zhongguancun, Beijing 100190, China; gaoyj20211@nanoctr.cn (Y.G.); panza2019@nanoctr.cn (Z.P.); jiaf2018@nanoctr.cn (F.J.); xucl2022@nanoctr.cn (C.X.); cuixy@nanoctr.cn (X.C.); 4 University of Chinese Academy of Sciences, Beijing 100049, China

## Error in the Figure

The authors regret that, in the published article [1], an error has been found within Figure 7; the H&E histological analysis of the lung after treatment with saline was incorrectly labeled. The corrected Figure 7 (H&E histological analysis of the lung after treatment with saline) is presented below:

The corrections made in this corrigendum do not affect the original conclusions. The authors apologize for any inconvenience or misunderstanding that this error may have caused. This correction was approved by the Academic Editor. The original publication has also been updated.

## Figures and Tables

**Figure 7:**
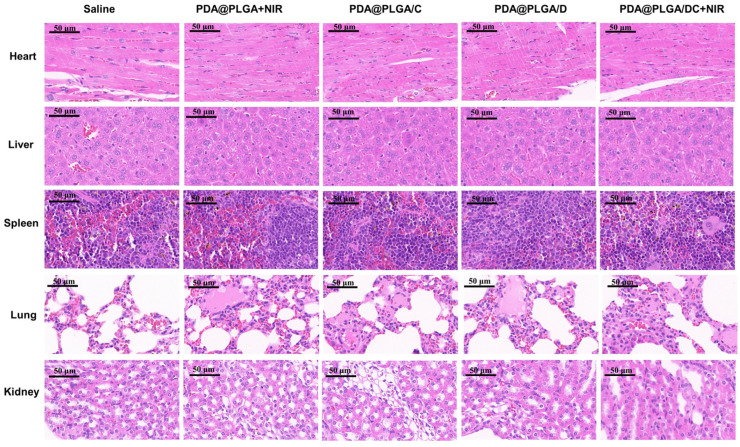
H&E histological analysis of major organs, including heart, liver, spleen, lung, and kidney after treatment with various formulations. Scale bars in all images represent 50 μm. Abbreviations: PDA@PLGA+NIR, PDA@PLGA NPs+NIR; PDA@PLGA/C, PDA@PLGA/C NPs; PDA@PLGA/D, PDA@PLGA/D NPs; PDA@PLGA/DC+NIR, PDA@PLGA/DC NPs+NIR.
