# Supplementary material for: Fabrication of Poly Dopamine@poly (Lactic Acid-Co-Glycolic Acid) Nanohybrids for Cancer Therapy via a Triple Collaboration Strategy
Source: Nanomaterials (Basel). 2023 Apr 24;13(9):1447. doi: 10.3390/nano13091447 (PMC10180254; doi:10.3390/nano13091447)
Supplement: Supplementary file 1 [file nanomaterials-13-01447-s001.zip › nanomaterials-2342820-supplementary.pdf]

Fabrication of Poly Dopamine@poly (Lactic Acid-Co-Glycolic Acid) Nanohybrids for Cancer Therapy via a Triple Collaboration Strategy

Yunhao Li [1,2,†], Yujuan Gao [3,4,†], Zian Pan [3,4], Fan Jia [3,4], Chenlu Xu [3,4], Xinyue Cui [3], Xuan Wang [3,*] and Yan Wu [3,4,*]

1 Department of Medicine, Li Ka Shing Faculty of Medicine, University of Hong Kong, Hong Kong, China; yunhaoli@connect.hku.hk
2 Department of General Surgery, Peking Union Medical College Hospital, Peking Union Medical College, Chinese Academy of Medical Sciences, Beijing 100730, China
3 CAS Key Laboratory for Biomedical Effects of Nanomaterials and Nanosafety, National Center for Nanoscience and Technology, No. 11 First North Road, Zhongguancun, Beijing 100190, China; gaoyj20211@nanoctr.cn (Y.G.)
4 University of Chinese Academy of Sciences, Beijing 100049, China
* Correspondence: wangxuan@nanoctr.cn (X.W.); wuy@nanoctr.cn (Y.W.)
† These authors contributed equally to this work.

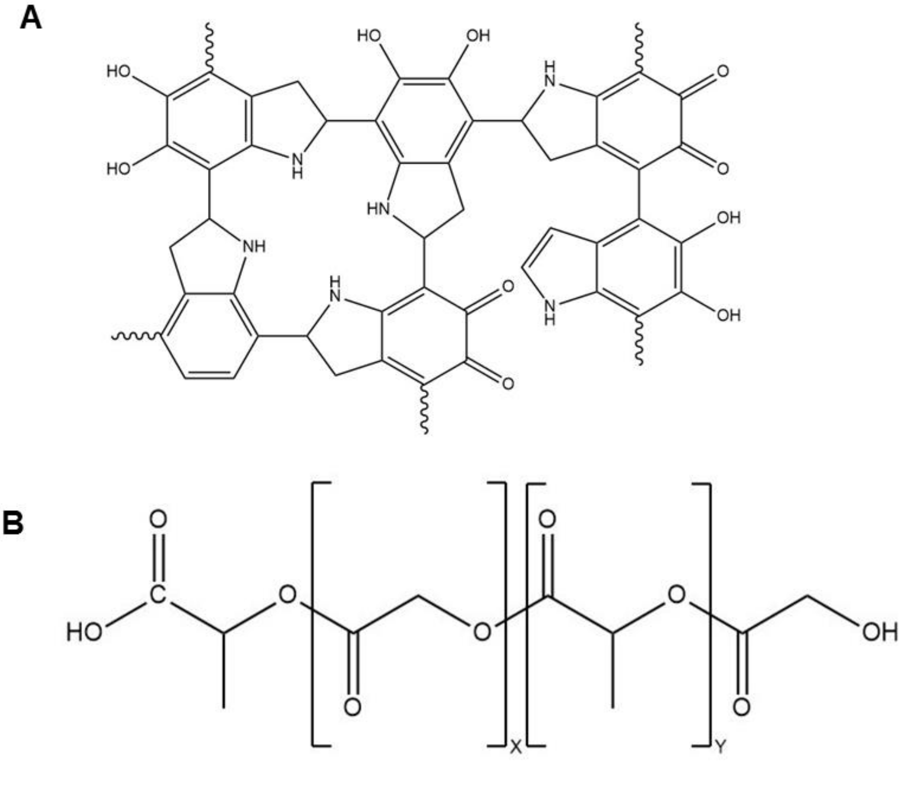

Figure S1. The structure PDA (A) and PLGA (B).

Table S1. Influence of the mass of DOX on encapsulation efficiency (EE) and loading content (LC) of PDA@PLGA/DC NPs.

PLGA (mg)	DOX (μg)	EE (%)	LC (%)
10	100	0	0
10	200	0	0
10	300	33	0.99
10	400	40	1.6
10	500	16.5	0.83

Table S2. Influence of the mass of CA4 on encapsulation efficiency (EE) and loading content (LC) of PDA@PLGA/DC NPs.

PLGA (mg)	CA4 (μg)	EE (%)	LC (%)
10	100	41.8	0.42
10	200	23.4	0.47
10	300	0	0
10	400	0	0
10	500	0	0

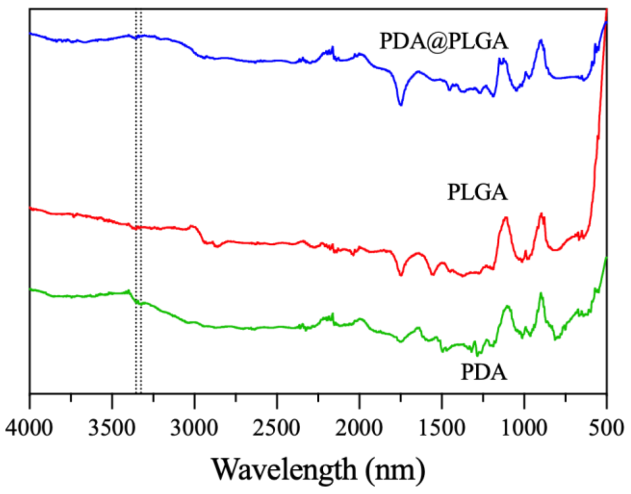

Figure S2. FT-IR spectra of PDA@PLGA NPs , PLGA NPs and PDA NPs.

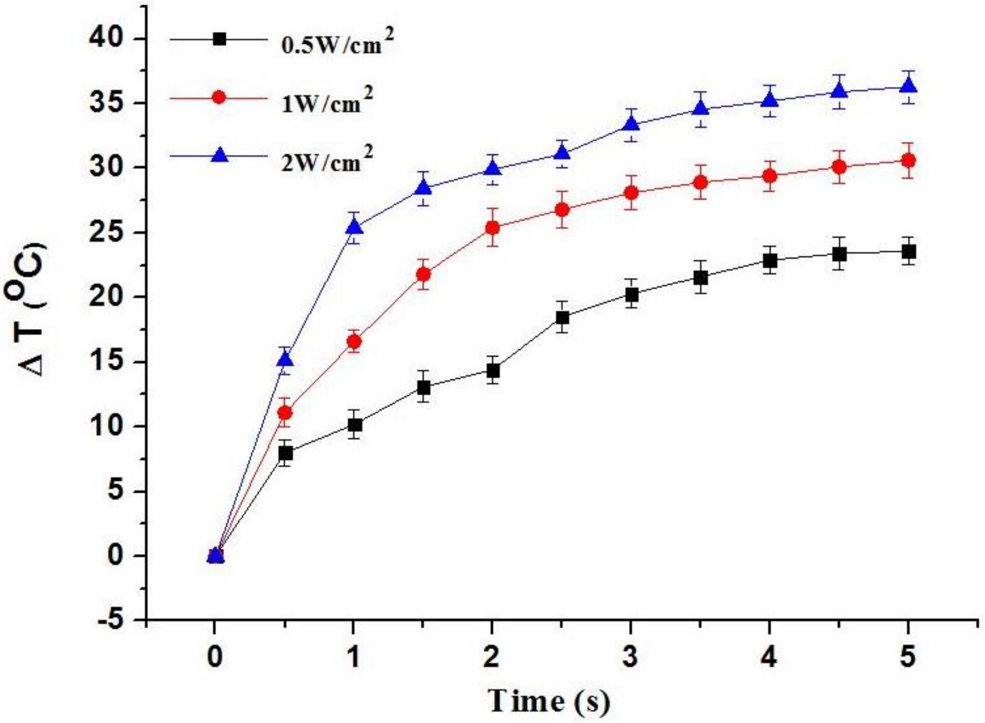

Figure S3. Temperature variation curves of PDA@PLGA/DC NPs at various power intensities with the same concentration at 150 μg/mL.

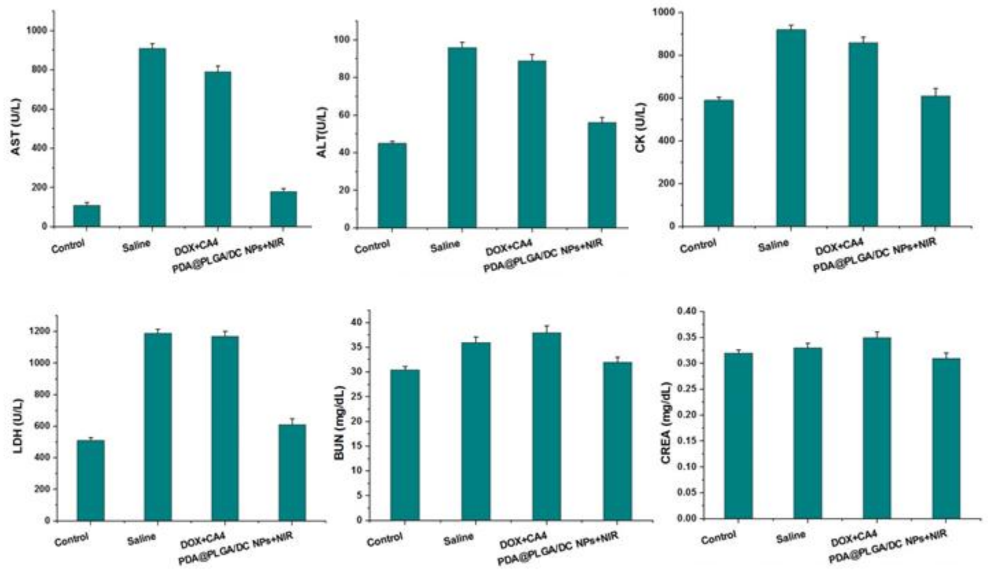

Figure S4. Serum biochemical examination of drug-loaded nanoparticles treated mice by saline, free DOX+CA4 and PDA@PLGA/DC NPs+NIR. Normal BALB/c mice served as the control group. Data were presented as mean± SD (n=5). Hepatic function indicators: AST, aspartate transaminase, ALT, alanine aminotransferase; Cardiac function indicators: CK, creatine kinase, LDH, lactate dehydrogenase; Renal function indicators: BUN, blood urea nitrogen, CREA, creatinine.
